# Nueva mutación en el gen *KMT2B* como causa de distonía generalizada de inicio temprano: reporte de caso

**DOI:** 10.7705/biomedica.6296

**Published:** 2022-09-02

**Authors:** Yully Andrea Rangel, Eugenia Espinosa

**Affiliations:** 1 Servicio de Neurología Pediátrica, Hospital Militar Central, Universidad Militar Nueva Granada, Bogotá, D.C., Colombia Universidad Militar Nueva Granada Hospital Militar Central Universidad Militar Nueva Granada Bogotá, D.C. Colombia

**Keywords:** discapacidad intelectual, distonía, estimulación cerebral profunda, enfermedades genéticas congénitas, trastornos distónicos, trastornos del movimiento, Intellectual disability, dystonia, deep brain stimulation, genetic diseases, inborn, dystonic disorders, movement disorders

## Abstract

La distonía por mutación en el gen *KMT2B* es un subtipo recientemente descrito del inicio focal de la enfermedad en los miembros inferiores que, posteriormente, evoluciona a una forma generalizada con compromiso cervical y orofaríngeo, disartria, trastorno secundario de la deglución y discapacidad intelectual.

Se describe el caso de una escolar de 10 años de edad, sin antecedentes de consanguinidad ni historia familiar de enfermedad neurológica, que presentó alteración de la marcha y distonía de inicio focal, de curso progresivo a una forma generalizada que afectó sus músculos orofaciales y bulbares con alteración significativa del lenguaje y la deglución. Los estudios metabólicos y sistémicos, incluidas las neuroimágenes, no evidenciaron anormalidades. Se hizo una secuenciación genómica completa y se identificó una nueva variante, probablemente patogénica heterocigota, en el gen *KMT2B*, la c.1205delC, p.(Pro402Hisfs*5), que causa desplazamiento en el marco de lectura. Este hallazgo explica el fenotipo de la paciente y la distonía de inicio temprano autosómica dominante.

Se reporta una nueva mutación heterocigota del gen *KMT2B* como causa de distonía generalizada de inicio temprano, no reportada en la literatura especializada hasta el momento. El diagnóstico de esta afección tiene implicaciones en el tratamiento y el pronóstico de los pacientes, porque las estrategias terapéuticas tempranas pueden prevenir su rápido deterioro y un curso más grave de la enfermedad.

## Abstract (en)

**Introduction:**

*KMT2B*-related dystonia is a recently described subtype of focal-onset dystonia in the lower limbs, evolving into a generalized form with cervical, oropharyngeal involvement, dysarthria, swallowing disorder and intellectual disability.

**Clinical case:**

We describe the case of a 10-year-old female patient, without a history of consanguinity or neurological disease. She manifested abnormal gait and dystonia with focal onset and progressive course with evolution into generalized dystonia, affecting orofacial and bulbar muscles, significant alteration of language and swallowing.

Metabolic and systemic studies, including neuroimaging, were found to be normal. A complete genomic sequencing study was performed identifying a new, probably pathogenic, heterozygous variant in the *KMT2B* gene, c.1205delC, p. (Pro402Hisfs*5), causing displacement in the reading frame, a finding that explains the patient’s phenotype and it is associated to autosomal dominant childhood-onset dystonia-28.

**Conclusion:**

We report a new heterozygous mutation in the *KMT2B* gene as a cause of generalized early-onset dystonia not reported in the literature until the date. The diagnosis of this pathology has implications for the treatment and prognosis of patients, given that therapeutic strategies implemented early can prevent the fast deterioration and severe course of this disease.

La distonía es el trastorno hipercinético más frecuente en pediatría. Se caracteriza por movimientos o posturas anormales repetitivas desencadenadas por contracciones musculares involuntarias. Su etiología es variable, aunque puede clasificarse como hereditaria o adquirida ^1^. Su aparición en la infancia temprana por mutaciones en el gen *KMT2B* es un subtipo de distonía generalizada recientemente descrito, el cual se inicia después de un neurodesarrollo normal, generalmente con distonía focal en miembros inferiores, y que compromete y altera progresivamente la calidad de vida del paciente en diversos grados ^2^.

Se describe aquí el caso de una paciente con distonía generalizada debido a una variante nueva, probablemente patogénica heterocigota, del gen *KMT2B*, con alteración de la marcha y distonía progresiva, disartria distónica y neuroimágenes normales.

## Caso clínico

Se presenta el caso de una escolar de 10 años de edad, nacida de padres no consanguíneos, sin historia familiar de enfermedades neurológicas, producto de la primera gestación; nació a las 36 semanas -con retardo del crecimiento intrauterino- mediante parto inducido y estuvo hospitalizada durante seis días por ictericia tratada con fototerapia.

Los patrones del desarrollo psicomotor fueron normales hasta los 3 años y 8 meses; posteriormente, presentó pérdida de hitos motores y del lenguaje, lo que se mantiene hasta la fecha. Inicialmente, presentó alteración de la marcha por inversión del pie izquierdo y, a los cuatro años, distonía en el miembro inferior izquierdo, la cual fue evolucionando progresivamente hasta comprometer los dos miembros inferiores y la marcha, por lo cual la paciente requirió silla de ruedas autopropulsada a partir de los siete años. El desarrollo del lenguaje receptivo y expresivo fue normal hasta el momento de inicio de la enfermedad, pero con el tiempo, desarrolló una disartria distónica que persiste hasta el momento.

Se trató inicialmente con levodopa-carbidopa con poco éxito, por lo que se adicionaron baclofeno y diazepam, lográndose una mejoría clínica parcial; durante los siguientes meses y años, se le administraron trihexifenidilo, biperideno y clonidina, y se le aplicó toxina botulínica sin mejoría significativa de la distonía.

Actualmente, la paciente está siendo tratada con levodopa-carbidopa, biperideno, trihexifenidilo, diazepam y clonidina, pero persisten la disartria distónica y el compromiso distónico orofaríngeo, y no se logra la sedestación ni la bipedestación; su distonía compromete principalmente los miembros inferiores.

Para el diagnóstico se hicieron estudios de patología y metabólicos completos, así como evaluaciones oftalmológica, cardiaca, radiológica, electrodiagnóstica y genética (cuadro 1). El estudio de secuenciación exómica no fue concluyente, sin embargo, en la secuenciación genómica completa se identificó una variante probablemente patogénica heterocigota en el gen *KMT2B*, la c.1205delC, p.(Pro402Hisfs*5), que causa desplazamiento del marco de lectura que se inicia en el codón 402. El nuevo marco de lectura culmina cuatro posiciones después de un codón de terminación. La variante fue confirmada por secuenciación Sanger.

**Cuadro 1:** Estudios practicados

Estudios	Prueba	Resultado
Metabólicos	- Aminoácidos cuantitativos en sangre por HPLC (*High Performance Liquid Chromatography*) - Estudios enzimáticos para enfermedad de Gaucher, creatina cinasa, lactato sérico, relación lactato- piruvato en plasma, ácido pirúvico, amonio, anticuerpos contra el virus linfotrópico humano de tipo 1 y 2, hexosaminidasa, betagalactosidasa, función hepática, función renal, función tiroidea, electrolitos, perfil óseo, tamizaje para enfermedad de Niemann Pick de tipo C.	Normal
Valoración oftalmológica		Normal
Neuroimágenes	Resonancia de columna, resonancia cerebral simple y espectroscopía cerebral	Normales
Electrodiagnóstico	Electromiografía y neuroconducciones normales	Normales
Cardiología	Ecocardiograma	Normales
Genéticos	Electrocardiograma Secuenciación del gen TOR1A Secuenciación exómica Secuenciación genómica completa	No se detectaron mutaciones puntuales, inserciones o deleciones en la región codificante y en las uniones exón-intrón. No se detectaron alteraciones. Variante probablemente patogénica heterocigota en el gen *KMT2B*: c.1205delC, p.(Pro402Hisfs*5)

### Consideraciones éticas

Se obtuvo autorización escrita del acudiente de la paciente para usar la información clínica y las pruebas diagnósticas descritas en esta publicación.

## Discusión

En el grupo de distonías de inicio temprano, la relacionada con mutaciones en el gen *KMT2B* presenta una prevalencia que oscila entre el 1,3 y el 38 %, con un rango de inicio de la sintomatología entre los 2 y los 7 años de edad ^1^. Hasta la fecha, se han reportado 39 casos de 16 familias ^2^. En la paciente de este informe, el inicio de la enfermedad fue temprano, hacia los tres años de edad, y no había antecedentes familiares de la enfermedad.

El mecanismo fisiopatológico de esta enfermedad no es claro. El gen *KMT2B* codifica la lisina metiltransferasa 2B, enzima que regula procesos de transferencia de grupos metilo al residuo 4 de la lisina de la histona H3, mediante lo cual cumple importantes funciones de expresión y transcripción génica ^2^. El gen se expresa en áreas de control motor del cerebro y el cerebelo, tanto en las etapas de desarrollo como en la adultez, y permite llevar a cabo procesos de mantenimiento de las funciones neuronales, de interconexión neuronal y de homeostasis tisular ^3^.

Clínicamente, se manifiesta en la primera década de la vida con alteraciones de la marcha, marcha en puntas, y, posteriormente, con distonía en miembros inferiores, hallazgo característico descrito en la literatura en más del 80 % de los casos ^2^. En el presente caso, en la paciente se inició con este síntoma característico en miembros inferiores y alteración progresiva de la marcha.

La distonía progresa hasta afectar de forma importante los músculos orofaciales y bulbares, comprometiendo de forma significativa la emisión de sonidos y la deglución ^3^.

Las infecciones, el estrés, las emociones y las intervenciones quirúrgicas pueden ser factores que exacerben la distonía causando deterioro clínico, lo cual fue evidente en esta paciente después de la intervención quirúrgica de corrección de la displasia de cadera y la subluxación, pues, aunque no presentó tormenta distónica, cursó con aumento de las distonías.

Se han descrito algunas características dismórficas asociadas con la enfermedad, por ejemplo, base nasal amplia, cara alargada, punta nasal bulbosa, clinodactilia o sindactilia, hipertricosis, talla baja, micrognatia y microcefalia ^1^, las cuales se relacionan con la longitud o el tamaño de la mutación. En el presente caso no se evidenciaron alteraciones dismórficas.

A nivel sistémico, se ha descrito compromiso cognitivo con retraso del neurodesarrollo, problemas de atención, retraso mental en diferentes grados, aplasia *cutis*, compromiso renal, estrabismo, astigmatismo o apraxia oculomotora, y, menos frecuentemente, síntomas psiquiátricos como ansiedad, depresión o trastorno obsesivo compulsivo ^1^. En el presente caso, la enfermedad cursó con hipercalciuria, sin embargo, los demás estudios descartaron compromiso cardiaco, renal, gastrointestinal, oftalmológico o metabólico. Actualmente, presenta un leve compromiso cognitivo y asiste a un colegio de inclusión.

En publicaciones recientes, se ha reportado la variabilidad en el fenotipo, con casos de *mioclonus*, movimientos coreiformes y disfunción cerebelosa asociada ^4^. Con menos frecuencia, se ha informado de portadores asintomáticos o con compromiso cognitivo sin distonía ^4^. En el caso descrito, se evidenció un fenotipo moderado. En algunos estudios se ha sugerido una correlación entre el genotipo y el fenotipo de la distonía por alteraciones en el *KMT2B*.

La variante encontrada en el presente caso produce una mutación de sentido erróneo, la cual se ha correlacionado con un inicio más tardío de la enfermedad (8 años) ^5^.

En el diagnóstico de esta enfermedad, la sospecha clínica es fundamental. Debe considerarse como posible causa en pacientes que presentan distonía con inicio focal en miembros inferiores, que luego se generaliza y compromete la musculatura orofacial y laríngea, con alteraciones secundarias del lenguaje o de la deglución, o en aquellos que cursan con distonía primaria y alguna o varias de las características dismórficas referidas anteriormente ^3^. Sin embargo, como factor importante de diferenciación debe considerarse la distonía sensible a levodopa, variedad Segawa, la cual mejora con dosis bajas de levodopa; en esta paciente, se inició la administración de levodopa sin obtener una reacción terapéutica adecuada.

Los estudios metabólicos y las neuroimágenes iniciales permiten descartar otras causas frecuentes de la distonía primaria y orientan el diagnóstico. La resonancia magnética cerebral puede sugerir algunas enfermedades que causan distonía en la infancia, como la enfermedad de Wilson, la encefalopatía mitocondrial, la deficiencia de piruvato deshidrogenasa o la aciduria orgánica, con base en el hallazgo del compromiso del núcleo basal en las secuencias de T2 o de susceptibilidad ^6^. En el presente caso, todos los estudios realizados inicialmente fueron normales.

La secuenciación exómica completa se sugiere como opción de primera línea en casos de distonía compleja o combinada por su buen rendimiento y efectividad en la detección de variantes asociadas ^7^. Se ha reportado una tasa de detección en distonías del 37,5 %, siendo mayor en la distonía de inicio en la infancia temprana ^8^.

En el caso descrito, esta prueba no fue concluyente, por lo que fue necesario hacer la secuenciación genómica, la cual detectó una variante patogénica asociada con la enfermedad, lo que se explica por la gran heterogeneidad genética de este grupo de enfermedades ^9^.

El manejo terapéutico de los movimientos anormales en pediatr**í**a incluye terapias modificadoras y sintomáticas, prevención de complicaciones secundarias y manejo de comorbilidades ^10^. En esta enfermedad, la mejoría observada en las diferentes series de casos ha sido variable, con escasa reacción ante la levodopa, lo que hace necesarios los tratamientos coadyuvantes ^9^.

Ha podido evidenciarse una mejor reacción terapéutica a los anticolinérgicos, como el trihexifenidilo en altas dosis (30-40 mg/día), sobre todo por mejoría en las habilidades motoras en edades más tempranas ^10^; el tratamiento conjunto con clonazepam ha demostrado ser una combinación efectiva ^3^. En el presente caso, la paciente ha recibido todos los medicamentos, incluso toxina botulínica, con poca mejoría.

La estimulación cerebral profunda ha demostrado beneficios en el tratamiento sintomático de trastornos del movimiento en pediatría, con una mejor evolución en las distonías primarias y mejores resultados cuanto más corto sea el curso de la enfermedad en el momento de la intervención ^11^. Las complicaciones son las inherentes a la intervención quirúrgica: problemas mecánicos del dispositivo, infecciones respiratorias, urinarias y del sistema nervioso central, o hematomas ^10^. La estimulación cerebral profunda bilateral del globo pálido interno ha permitido mejorar la deambulación independiente, con mejor reacción terapéutica en pacientes con distonías que aparecen a menor edad, que logran estabilizarse en el mediano y largo plazo ^3^^,^^11^. Esta paciente fue remitida a neurocirugía funcional con el fin de establecer opciones terapéuticas adicionales ^3^^,^^11^.

## Conclusión

Se reporta una nueva mutación heterocigota en el gen *KMT2B* como causa de distonía generalizada de inicio temprano, no informada en la literatura científica hasta el momento. Para lograr un diagnóstico adecuado, se deben evaluar los hallazgos clínicos y paraclínicos, siendo fundamental el estudio genético en los casos en los que se detectan alteraciones, lo cual tiene implicaciones en el tratamiento y el pronóstico de los pacientes, ya que las estrategias terapéuticas implementadas tempranamente pueden contribuir a disminuir la gravedad de los síntomas.
